# Varenicline and Nicotine Replacement Use Associated With US Food and Drug Administration Drug Safety Communications

**DOI:** 10.1001/jamanetworkopen.2019.10626

**Published:** 2019-09-04

**Authors:** Ravi J. Desai, Meghan M. Good, Alvaro San-Juan-Rodriguez, Andrew Henriksen, Francesca Cunningham, Inmaculada Hernandez, Chester B. Good

**Affiliations:** 1 VA Center for Medication Safety, Veterans Health Administration, Pittsburgh, Pennsylvania; 2 Department of Pharmacy and Therapeutics, School of Pharmacy, University of Pittsburgh, Pittsburgh, Pennsylvania; 3 VA Center for Medication Safety, Veterans Health Administration, Hines, Illinois; 4 Centers for High-Value Health Care and Value Based Pharmacy Initiatives, Insurance Services Division, University Pittsburgh Medical Center Health Plan, Pittsburgh, Pennsylvania

## Abstract

**Importance:**

Drug safety communications released by the US Food and Drug Administration (FDA) are often based on limited evidence on safety signals after approval. Varenicline may serve as a relevant case study because it was the target of several FDA communications in 2008 and 2009; ultimately, the Evaluating Adverse Events in a Global Smoking Cessation Study (EAGLES) dismissed safety concerns on increased suicidal thoughts and aggressive and erratic behavior on December 16, 2016.

**Objective:**

To examine the association between FDA drug safety communications and the use of varenicline.

**Design, Setting, and Participants:**

Retrospective, longitudinal, cross-sectional study of Veterans Health Administration (VHA) outpatient data from October 1, 2001, through December 31, 2018, and Medicaid drug state use data from July 1, 2006, through September 30, 2018, on varenicline prescribing.

**Main Outcomes and Measures:**

Prescribing records for varenicline and nicotine replacement therapy (NRT) in the VHA were extracted, and the number of unique varenicline and NRT users in the VHA per quarter was measured. An interrupted time series analysis was performed to describe the association between FDA safety warnings and the use of varenicline and NRT. To test the generalizability of the findings, similar analyses were conducted using the number of prescriptions reimbursed for varenicline by Medicaid every quarter in 2006-2018.

**Results:**

After its addition to the VHA national drug formulary in January 2007, varenicline use presented a steady increase, reaching a peak of 32 581 quarterly unique users in the first quarter of 2008. Within 12 months of the February 1, 2008, public health advisory, quarterly varenicline use in VHA patients decreased by 68.7% (from 32 581 to 10 182 patients; *P* < .001 for slope change), and NRT use increased by 32.1% (from 55 728 to 73 629 patients; *P* < .001 for slope change). In Medicaid prescriptions, varenicline use decreased by 38.0% (from 109 308 to 67 761 prescriptions; *P* < .001 for slope change) within 12 months of the 2008 public health advisory. Twelve months after the publication of the EAGLES trial, which showed no significant increase in psychiatric/behavioral effects with varenicline relative to NRT, use of varenicline increased by 42.7% in VHA patients (from 9251 to 13 199 patients; *P* = .01 for slope change) and by 26.0% in Medicaid prescriptions (112 063 to 141 122; *P* = .26 for slope change ).

**Conclusions and Relevance:**

With use of varenicline as a case study, early communications from the FDA and VHA followed by a labeling change appeared to be associated with a considerable decrease in drug use, which may have been associated with negative public health consequences.

## Key Points

**Question:**

Did use of varenicline change after early US Food and Drug Administration drug safety communications regarding neuropsychiatric adverse events?

**Findings:**

This cross-sectional study observed a reduction in varenicline prescribing following the release of US Food and Drug Administration drug safety communications on the medication. Interrupted time series analysis showed a 68.7% decrease in Veterans Health Administration outpatient prescriptions and a 38.0% decrease in Medicaid prescriptions.

**Meaning:**

In the wake of US Food and Drug Administration and Veterans Health Administration communications, prescriptions for varenicline decreased significantly, which may have been associated with negative public health consequences.

## Teaser

This cross-sectional study uses data from the Veterans Health Administration and Medicaid to examine changes in varenicline prescribing from the time of US Food and Drug Administration (FDA) approval through publication and removal of public health advisories.

## Introduction

The US Food and Drug Administration (FDA) drug safety communications and public health advisories are intended to inform the public about emerging drug-related safety issues.^1^ These communications are frequently based on early safety signals that require ongoing assessment for confirmation but nonetheless can substantially affect drug use with potentially unintended consequences.^2,3^ The FDA recognizes this concern and recently commissioned a study to evaluate postmarket FDA safety labeling changes.^4^ That report developed a framework for future studies to inform the FDA on the outcome of safety labeling changes.

Varenicline was approved by the FDA in May 2006. On November 20, 2007, the FDA released the first safety communication regarding suicidal thoughts and aggressive and erratic behavior, which was followed by a public health advisory on February 1, 2008, and the addition of a boxed warning on July 1, 2009.^5^ In June 2016, the Evaluating Adverse Events in a Global Smoking Cessation Study (EAGLES) was published, showing no significant increase in psychiatric/behavioral effects with varenicline relative to nicotine replacement therapy (NRT) or placebo.^6^ Varenicline’s boxed warning was removed on December 16, 2016.

The Veterans Health Administration (VHA) has recognized the importance of smoking in health-related outcomes and actively promotes efforts to identify smokers and offer treatment. By 1998, more than 90% of smokers in the VHA were actively offered treatment; by 2007, the VHA adopted smoking cessation performance measures that encouraged offering smoking cessation drugs and referral for intensive smoking cessation programs.^7^ As one of the treatments available to meet such performance metrics, varenicline’s availability was accepted widely within the VHA.

In this study, we aimed to evaluate the association between FDA drug communications and the use of varenicline within the VHA and evaluate the generalizability of our findings using Medicaid data. We also simulated the potential consequences of decreased varenicline use on lost opportunities to assist patients to quit smoking and their downstream health outcomes.

## Methods

### Use of Varenicline and NRT in the VHA

We extracted data on outpatient prescriptions between October 1, 2001, and December 31, 2018, from the VHA Pharmacy Benefits Management Services. We identified the number of unique patients receiving varenicline and/or NRT every quarter. Patients who received medications in more than 1 quarter were counted in each period. This study followed the Strengthening the Reporting of Observational Studies in Epidemiology (STROBE) reporting guideline for cross-sectional studies.^8^ This study was deemed exempt by the VHA Pittsburgh Institutional Review Board and informed consent was not required owing to the use of aggregate, unidentifiable data.

### Use of Varenicline in Medicaid

We extracted all fee-for-service and managed care records for varenicline from July 1, 2006, the second quarter (Q2) of 2006 (varenicline approval), to September 30, 2018, the Q3 of 2018 (most recent data at the time of analysis), which contained the number of units, number of prescriptions, and total amount reimbursed in each state and quarter for a given product. We calculated the number of prescriptions filled for varenicline nationwide every quarter.

### Estimated Health Outcomes 

To assess the potential outcome of lost opportunities for successful smoking cessation with the use of varenicline, we used data from several observational studies. To estimate the additional use of varenicline vs NRT for long-term smoking cessation in a real-world primary care setting, we used a rate of 4.99 per 100 successful long-term smoking cessation attempts using varenicline from a large study of more than 220 000 patients.^9^ To estimate the outcome of sustained smoking cessation on mortality, we used data from a study of successful quitters in a smoking cessation program that examined mortality at a mean of 14.5 years after intervention.^10^ We assumed that without safety warnings for varenicline, its use in the VHA would have remained stable in 2009-2016 at the 2008 rate. This estimation is conservative since drug adoption often takes 2 to 3 years; thus, it is likely that use trend would have continued to increase.^11^ We calculated the difference between actual varenicline use through 2016, when the warnings were lifted. In addition, we estimated deaths due to lost smoking cessation opportunities over the 8 years of varenicline warnings.

### Statistical Analysis

To formally test the association between safety communications and varenicline and NRT use in the VHA, we divided the study into 3 periods and constructed an interrupted time series analysis with a linear regression model. The model regressed the number of unique patients against a continuous variable for time (quarter), 2 indicator variables for the 3 periods of interest, the second-order interactions between them, an indicator variable for NRT, and the second- and third-order interactions between the time and periods variables and this indicator. The first period corresponded to the time from the beginning of the study until the publication of the FDA public health advisory on varenicline (October 1, 2001, to February 1, 2008), the second period started with the publication of this health advisory and ended with the removal of the boxed warning (February 1, 2008, to December 16, 2016), and the third period corresponded to the time after the removal of the boxed warning (December 16, 2016, to December 31, 2018).

In addition, we duplicated interrupted time series analyses using Medicaid state drug use data to assess the generalizability of changes in varenicline use within the VHA. For Medicaid use data, interrupted time series analyses were constructed as described above but included only varenicline records, used the number of prescriptions instead of the number of patients as the outcome, and began the first period with varenicline approval in Q2 2006. All *P* values were from 2-sided tests, and results were deemed statistically significant at *P* < .05. Statistical analyses were conducted with SAS, version 9.4 (SAS Institute Inc).

## Results

### Trends in Varenicline and NRT Use in the VHA

**Figure 1.**
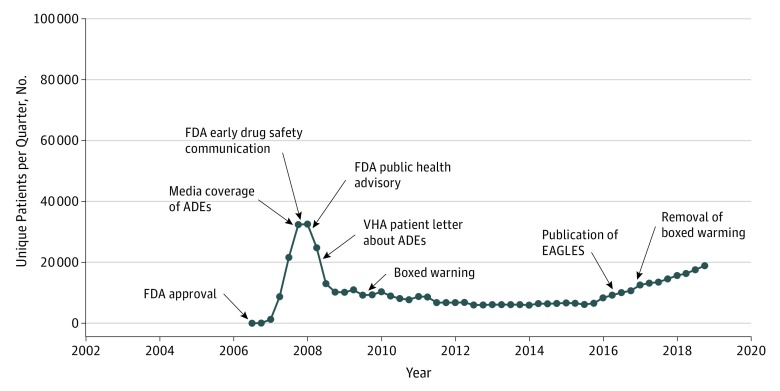
Timeline of Adverse Drug Events (ADEs) and Varenicline Use in the Veterans Health Administration (VHA), July 1, 2006, to December 31, 2018 EAGLES indicates Evaluating Adverse Events in a Global Smoking Cessation Study; FDA, US Food and Drug Administration.

**Figure 2.**
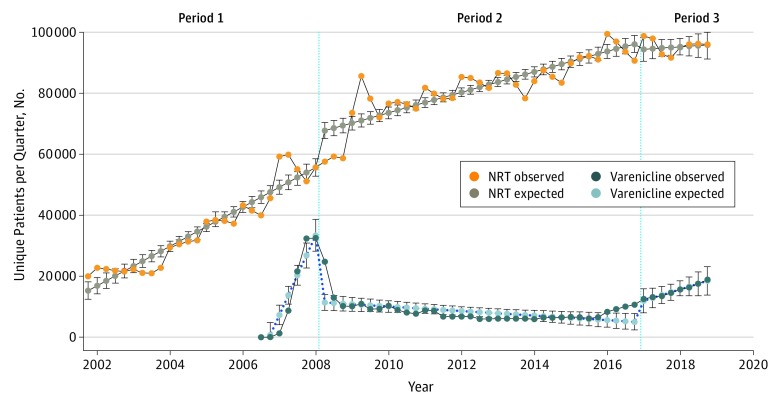
Trends in Varenicline and Nicotine Replacement Therapy (NRT) Use in the Veterans Health Administration, October 1, 2001, to December 31, 2018. Number of unique patients using varenicline and NRT every quarter in the Veterans Health Administration. Values were estimated with interrupted time series analyses. Period 1 indicates the time before the publication of the US Food and Drug Administration (FDA) public health advisory on February 1, 2008; period 2, the time between the publication of the FDA public health advisory and the removal of the boxed warning on December 16, 2016; and period 3, the time after the removal of the boxed warning.

After its addition to the VHA national drug formulary in January 2007, varenicline use presented a steady increase, reaching a peak of 32 581 quarterly unique users in Q1 2008 (Figure 1). Within 12 months of the 2008 FDA public health advisory, quarterly varenicline use decreased by 68.7% (from 32 581 to 10 182 patients; *P* < .001 for slope change) (Figure 2), while NRT use increased by 32.1% (from 55 728 to 73 629 patients; *P* < .001 for slope change). Over the 12 months following the addition of a boxed warning on July 1, 2009, quarterly varenicline use declined an additional 18.5% (from 10 980 to 8946 patients). Varenicline use reached a nadir in early 2014, when the number of unique quarterly users was 5990, representing an 81.6% decline from Q1 2008. Twelve months following the publication of EAGLES in June 2016,^6^ quarterly varenicline use increased by 42.7% (from 9251 to 13 199 patients; *P* = .01 for slope change). By Q4 2018, the number of unique quarterly varenicline users had increased to 18 909. From 2008 to 2018, quarterly NRT users increased by 72.5% (from 55 728 to 96 103 patients).

### Trends in Varenicline Use in Medicaid

**Figure 3.**
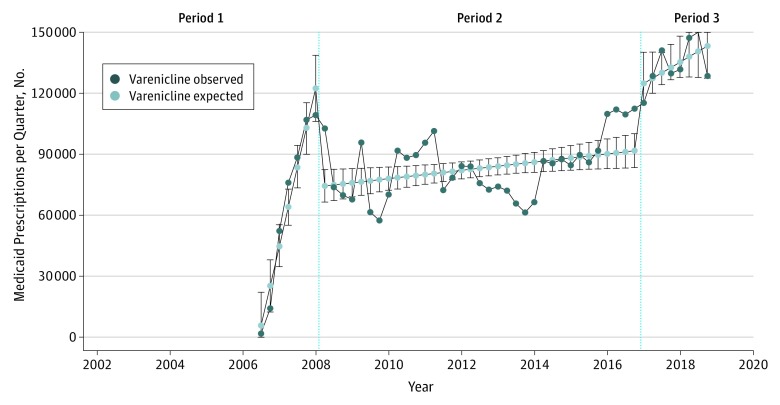
Trends in Varenicline Use in Medicaid Prescriptions, October 1, 2001, to December 31, 2018 Number of varenicline prescriptions every quarter in Medicaid. Values were estimated with interrupted time series analyses. Period 1 indicates the time before the publication of the US Food and Drug Administration (FDA) public health advisory on February 1, 2008; period 2, the time between the publication of the FDA public health advisory and the removal of the boxed warning on December 16, 2016; and period 3, the time after the removal of the boxed warning.

Varenicline use in Medicaid experienced a similar increase during the first year after its approval, reaching a peak of 109 308 prescriptions in Q1 2008 (Figure 3). In the year following the 2008 public health advisory, varenicline use in Medicaid decreased by 38.0% (from 109 308 to 67 761 prescriptions; *P* < .001 for slope change). After this substantial decrease, trends in varenicline use fluctuated, starting to increase toward the beginning of 2014. Twelve months following the publication of EAGLES,^6^ varenicline use increased by 26.0% (from 112 063 to 141 122 patients; *P* = .26 for slope change). Varenicline use in Medicaid peaked in Q2 2018, reaching 150 067 prescriptions.

### Estimated Health Outcomes 

If varenicline use in the VHA had remained stable between 2009 and 2016, we estimated that 411 712 more VHA beneficiaries would have been treated with varenicline during that time. Assuming a quit rate of 4.99% per year,^9^ an estimated additional 20 544 patients did not quit smoking in this period, which corresponds to 89 580 additional patient smoking years in 2009-2016. The number of patients who did not quit smoking owing to decreased varenicline use in the VHA was estimated to result in a potential of 454 deaths.

## Discussion

Using varenicline as a case study, we found that FDA drug safety communications were followed by significant reductions in drug use, with what may be resultant lost opportunity for successful smoking cessation. Because varenicline has been shown to be more effective than other pharmacotherapy options in assisting quit attempts in both clinical trials and real-world situations,^12^ it is likely that the FDA warnings regarding varenicline safety and resultant actions of health care organizations and prescribing clinicians led to a net health care loss.

Our estimates of a potential of 454 lost lives within the VHA might be viewed as speculative owing to multiple assumptions used in our analysis. We are unaware of any prospective data comparing mortality benefit of successful smoking cessation between varenicline and NRT. One short-term observational study in the United Kingdom with 2 years of follow-up did not find any difference in mortality or hospitalization between varenicline and NRT users.^13^ Conversely, another study using national claims databases in the United States reported fewer smoking-attributable outcomes and lower health care costs in patients receiving varenicline compared with NRT, although mortality was not reported.^14^ We believe that, while the magnitude of mortality estimates are uncertain, the overall effect on public health is probably conservative, as we did not assume an increase in use of varenicline as would be suggested by the rapid uptake of the medication from 2006 to 2008. Moreover, we only estimated its association with mortality and only during the limited period of observation. Smoking cessation decreases nonfatal myocardial infarction, stroke, chronic pulmonary disease, and other significant health outcomes,^15^ particularly in high-risk patient populations, such as the VHA. Given that varenicline is significantly more effective than NRT for smoking cessation,^12^ the decreased use of varenicline following warnings could be expected to have a significant association with public health.

Prior studies evaluating FDA safety communications and labeling changes for other medications have reported a significant association with use. For instance, in October 2003, the FDA released a public health advisory noting a risk of suicidality associated with antidepressant use in pediatric populations.^16^ In October 2004, a boxed warning for risk of suicidality was mandated for antidepressants used in children and adolescents based on limited data.^17^ Following the release of the public health advisory and boxed warning, prescribing rates of antidepressants in children and adolescents decreased from 4.1 million prescriptions in 2002 to 2003 to 2.8 million from 2006 to 2007.^16^ This decrease in prescribing of antidepressants in younger patients following FDA warnings was especially concerning because, in 2006, suicide was the third leading cause of death in younger patients, and only 2% of this population committing suicide were receiving any antidepressant medication therapy at the time of their death.^18^ Our results may build on this body of literature that demonstrates the association between FDA communications and drug use.

We recognize that part of the FDA’s responsibility for the safe and effective use of medications requires assessment of early signals for adverse clinical outcomes associated with use of medications after approval. These early warnings are released to inform clinicians and the public about the potential for drug-related safety concerns prior to conclusive evidence for causality and often describe the occurrence of less common outcomes that were not identified in clinical trials owing to small sample sizes or limited follow-up. The VHA uses these FDA reports to examine potential adverse outcomes in its covered population and in some cases may develop communications to send to VHA clinicians advising them of potential drug safety concerns.

The VHA response to FDA warnings was likely more intense than in most other health care settings. Varenicline use in the VHA was the target of much attention when, in 2008, the Committee on Veterans Affairs held a congressional hearing titled “Why Does the US Department of Veterans Affairs Continue to Give a Suicide-Inducing Drug to Veterans With Post Traumatic Stress Disorder?”^19^ This hearing, which was stimulated by a series of reports in the *Washington Times*, focused on a clinical trial funded by the VHA (CSP 519) that included varenicline as part of a smoking cessation intervention for veterans with posttraumatic stress disorder.^20^ The VHA was accused of a lack of response to the FDA communications on safety concerns with varenicline, and concern was raised that the VHA was experimenting on veterans by offering varenicline as part of a larger smoking cessation intervention. Subsequent internal VHA concerns regarding the safety of varenicline in patients with mental illness, combined with FDA safety warnings, media reports, and congressional concern, triggered a series of reiterated safety warnings by the VHA and changes in their varenicline prescribing criteria. Specifically, the VHA established requirements to formally assess veterans for suicide risk prior to starting varenicline therapy, as well as to obtain ongoing risk assessments if the medication was prescribed.^21^ The VHA was particularly concerned about the safety of varenicline treatment for patients with underlying mental illness. In addition to changes in prescribing criteria, the VHA developed an ongoing varenicline safety surveillance program and reviewed observational data from veterans who received varenicline, including those with and without serious mental illnesses. These additional VHA warnings, prescribing guidance changes, and ongoing safety initiatives likely explain the observation that the decrease in use of varenicline was more substantial in the VHA population than in Medicaid.

In the era of accelerated drug approvals, it is particularly important to reexamine the evidence necessary for approval as well as the evidence base for the release of postmarket safety concerns or for the identification of lack of effectiveness. It is estimated that, prior to approval, a drug is studied in a range from a few hundred to 3000 individuals.^22,23^ In the case of varenicline, FDA approval was based on a total of 4690 patients enrolled in clinical trials; however, most of these individuals were enrolled in short-term studies of 12 weeks.^24^ With no signal for suicide or serious adverse neuropsychiatric events identified in clinical trials, the FDA faced a dilemma when early reports of serious neuropsychiatric events in patients exposed to varenicline emerged. This difficult situation led to several drug safety communications and, ultimately, a boxed warning, which remained until subsequent clinical trials and studies—including a varenicline safety study performed by the VHA Center for Medication Safety^25^—did not confirm these safety signals. Although we acknowledge the importance of drug safety communications and understand the need to respond with label changes with a goal to protect the public as drug safety signals are being evaluated, the FDA and health care organizations, such as the VHA, need to balance safety concerns with the possibility that even preliminary warnings can have significant implications for prescribing. While alerting the public about potential drug-related harms, these communications can also have unintended consequences and may lead to poorer health outcomes by preventing patients from benefiting from effective medications.

Given the uncertain nature of early safety signals, it will be important to reexamine the evidence base necessary to release safety communications and ponder it against the strength of the evidence collected from preapproval clinical trials. Conversely, although our report and previous reports have focused on unintended adverse effects of these FDA communications, we acknowledge the likelihood of positive clinical outcomes associated with these early communications. Future investigations should examine both positive and negative consequences of these drug safety communications.

### Limitations

Our study is subject to several limitations. Our findings of significant changes in varenicline prescribing using an observational study design may not be generalizable to other patient populations. However, we observed a significant decrease of 38.0% in varenicline use in Medicaid, which is consistent with prior reports from Pfizer that documented a decrease in sales of approximately one-third in varenicline use from 2008 to 2014.^26^ Our projections of lives lost were derived from non-VHA patient populations and thus might not be applicable to our patient population. The greatest limitation is that, for VHA varenicline slopes, we were unable to differentiate prescribing changes associated with FDA warnings and labeling changes from those associated with VHA drug safety communications and changes in prescribing guidance and national temporal trends.

## Conclusions

This study found that early communications from the FDA followed by a labeling change were associated with additional safety measures within the VHA and a subsequent decrease in prescribing of varenicline for smoking cessation, which may have been associated with negative public health consequences. Analyses from other health care systems should evaluate the association between early drug safety communications and medication prescribing and potential benefits or harms that might result from those changes. In responding to these early communications, health care systems should consider the magnitude of potential harm against known benefit from use of that medication.
